# Supplementary material for: The Systems Biology Research Tool: evolvable open-source software
Source: BMC Syst Biol. 2008 Jun 29;2:55. doi: 10.1186/1752-0509-2-55 (PMC2446383; doi:10.1186/1752-0509-2-55)
Supplement: Additional file 1 — SBRT Archive. An archive of the current version of the Systems Biology Research Tool. [file 1752-0509-2-55-S1.zip › sbrt-1.4.0/doc/developers_guide/api/sbrt/kernel/io/package-tree.html]

sbrt.kernel.io Class Hierarchy


|  |  |  |
| --- | --- | --- |
| **Overview** \| **Package** \| Class \| Use \| **Tree** \| **Deprecated** \| **Index** \| **Help** | |  |
| **PREV**   **NEXT** | **FRAMES**    **NO FRAMES**     **All Classes** |  |


---


## Hierarchy For Package sbrt.kernel.io

**Package Hierarchies:**: All Packages

---

## Class Hierarchy

- java.lang.Object
  - sbrt.kernel.io.**AbstractResultsManager**<R> (implements sbrt.kernel.io.ResultsManager<T>)
    - sbrt.kernel.io.**ListResultsManager**<R> (implements java.lang.Iterable<T>)- sbrt.kernel.io.**SingleRm**<R>
    - java.lang.Throwable (implements java.io.Serializable)
      - java.lang.Exception
        - java.lang.RuntimeException
          - sbrt.kernel.io.**ConflictingInputException**
      - sbrt.kernel.io.**WarningHandlerFactory**

## Interface Hierarchy

- sbrt.kernel.io.**ResultsManager**<T>- sbrt.kernel.io.**WarningHandler**

---


|  |  |  |
| --- | --- | --- |
| **Overview** \| **Package** \| Class \| Use \| **Tree** \| **Deprecated** \| **Index** \| **Help** | |  |
| **PREV**   **NEXT** | **FRAMES**    **NO FRAMES**     **All Classes** |  |


---
